# What Would Jaws Do? The Tyranny of Film and the Relationship between Gaze and Higher-Level Narrative Film Comprehension

**DOI:** 10.1371/journal.pone.0142474

**Published:** 2015-11-25

**Authors:** Lester C. Loschky, Adam M. Larson, Joseph P. Magliano, Tim J. Smith

**Affiliations:** 1 Department of Psychological Sciences, Kansas State University, Manhattan, KS, United States of America; 2 Department of Psychology, University of Findlay, Findlay, OH, United States of America; 3 Department of Psychology, Northern Illinois University, DeKalb, IL, United States of America; 4 Department of Psychology, Birkbeck University of London, London, United Kingdom; University of Melbourne, AUSTRALIA

## Abstract

What is the relationship between film viewers’ eye movements and their film comprehension? Typical Hollywood movies induce strong attentional synchrony—most viewers look at the same things at the same time. Thus, we asked whether film viewers’ eye movements would differ based on their understanding—the *mental model* hypothesis—or whether any such differences would be overwhelmed by viewers’ attentional synchrony—the *tyranny of film* hypothesis. To investigate this question, we manipulated the presence/absence of prior film context and measured resulting differences in film comprehension and eye movements. Viewers watched a 12-second James Bond movie clip, ending just as a critical predictive inference should be drawn that Bond’s nemesis, “Jaws,” would fall from the sky onto a circus tent. The No-context condition saw only the 12-second clip, but the Context condition also saw the preceding 2.5 minutes of the movie before seeing the critical 12-second portion. Importantly, the Context condition viewers were more likely to draw the critical inference and were more likely to perceive coherence across the entire 6 shot sequence (as shown by event segmentation), indicating greater comprehension. Viewers’ eye movements showed strong attentional synchrony in both conditions as compared to a chance level baseline, but smaller differences between conditions. Specifically, the Context condition viewers showed slightly, but significantly, greater attentional synchrony and lower cognitive load (as shown by fixation probability) during the critical first circus tent shot. Thus, overall, the results were more consistent with the tyranny of film hypothesis than the mental model hypothesis. These results suggest the need for a theory that encompasses processes from the perception to the comprehension of film.

## Introduction

A key question in research on visual attention is the degree to which eye movements are influenced by higher-level cognitive processes [1–6]. Many people might assume that movie viewers’ eye movements are affected by their moment-to-moment comprehension processes as they watch films or TV, but surprisingly, there is little research exploring this everyday experience. Suppose that two people watching the same movie have very different higher-level understandings of it while watching it, as reflected by their predictions of what will happen next in the story. Will their eye movements show corresponding differences or will they be largely the same? The answer to this question has important implications for theories of attention and comprehension, and for real-world applications in mass media and education.

Over the last 30 years, a critically important question in theories of scene perception has been the degree to which higher-level cognition (*endogenous* factors) versus visual stimulus attributes (*exogenous* factors) drive attention and eye movements when viewing scenes. Studies of exogenous influences on eye movements during static scene viewing have shown that computational models of visual saliency (using visual feature contrast) can predict where viewers fixate in scenes at above chance levels [7–9]. However, when the compared to the endogenous effects of a viewing task (i.e., the viewers’ goals), eye movement studies have consistently shown far stronger *endo*genous effects than *exo*genous effects [3–5, 10, 11]. Furthermore, such effects of viewing task have been recently found in unedited natural videos of dynamic scenes [12]. Thus, if one asks what controls viewers’ eye movements when looking at either static or dynamic scenes, the weight of evidence is clearly tilted in the direction of attention being more under endogenous control by cognition than under exogenous control by features of the visual scene. However, such studies have not used visual scenes that are specifically designed to direct viewer attention such as in narrative film.

There is a fledgling body of research on film perception and comprehension, which builds largely on studies of reading comprehension (e.g., [13, 14]) and scene perception (e.g., [15] for review, see [16]. Reading research has a richer history of using eye movements to index higher-level comprehension processes. The “eye-mind hypothesis” is a fundamental theoretical assumption linking cognitive processes with eye movements (e.g., [17–19]). It states that moment-to-moment changes in where and when the eyes move are directly linked to on-line comprehension processes. Studies investigating this hypothesis have shown both clear effects of higher order cognition on eye movements (for review, see [20]) but also a degree of independence between them [21–23]. One might expect that the same would hold true in scene perception. However, to date, no such studies have investigated the relationship between viewers’ eye movements and their *comprehension* of narrative events.

A key finding in studies of movie viewers’ eye movements has been overwhelming evidence of *attentional synchrony*—namely, different viewers tendency to look at the same things on the screen at the same times [12, 24–26]. This may be because certain low-level features of *Hollywood style* films strongly attract attention [9, 25]. Over the past 70 years of filmmaking, films have evolved toward increasingly shorter shot lengths (i.e., more frequent cuts), more motion in the frame, and higher contrast between dark and light regions [16, 27, 28]. All of these changes more effectively direct viewers’ attention, which Bordwell [29] calls the *intensified continuity* film-making style. Thus, attentional synchrony across viewers is likely due to exogenous control of viewers’ attention and eye movements. If so, there may be little room for viewers to exert endogenous (cognitive) control of their eye movements while trying to comprehend films. We will call this the “*tyranny of film”* to suggest that there may be little room for individual differences in how viewers visually attend to a film.

Importantly, a large body of research has shown that *where* people look, and thus attend to, strongly affects *what* they are aware of and later remember (e.g., [30–32]), producing the phenomena of change blindness and inattentional blindness (though some studies have shown change blindness or inattentional blindness for objects at or near fixation [33, 34]). This has led to extensive research on *where* and *when* people look in scenes. Together, the phenomena of attentional synchrony in film viewing and the importance of attention for awareness and memory for scene contents, suggests that most viewers of Hollywood style films will have a shared awareness and memory for the contents of those films. In fact, most viewers generate similar inferences and mental models for narrative films, in part driven by the use of cinematic conventions [35, 36]. Thus, the tyranny of film may extend beyond attention and eye movements to encompass awareness, comprehension, and memory, suggesting that film exerts a degree of mind control (for viewers' correlated brain activity while watching Hollywood films, see [37]). Such evidence would help explain the importance of film for propaganda [38, 39], and the incredible amounts of money spent on televised advertising, projected to reach $81.6 Billion per year in the US by 2017 [40].

**Fig 1:**
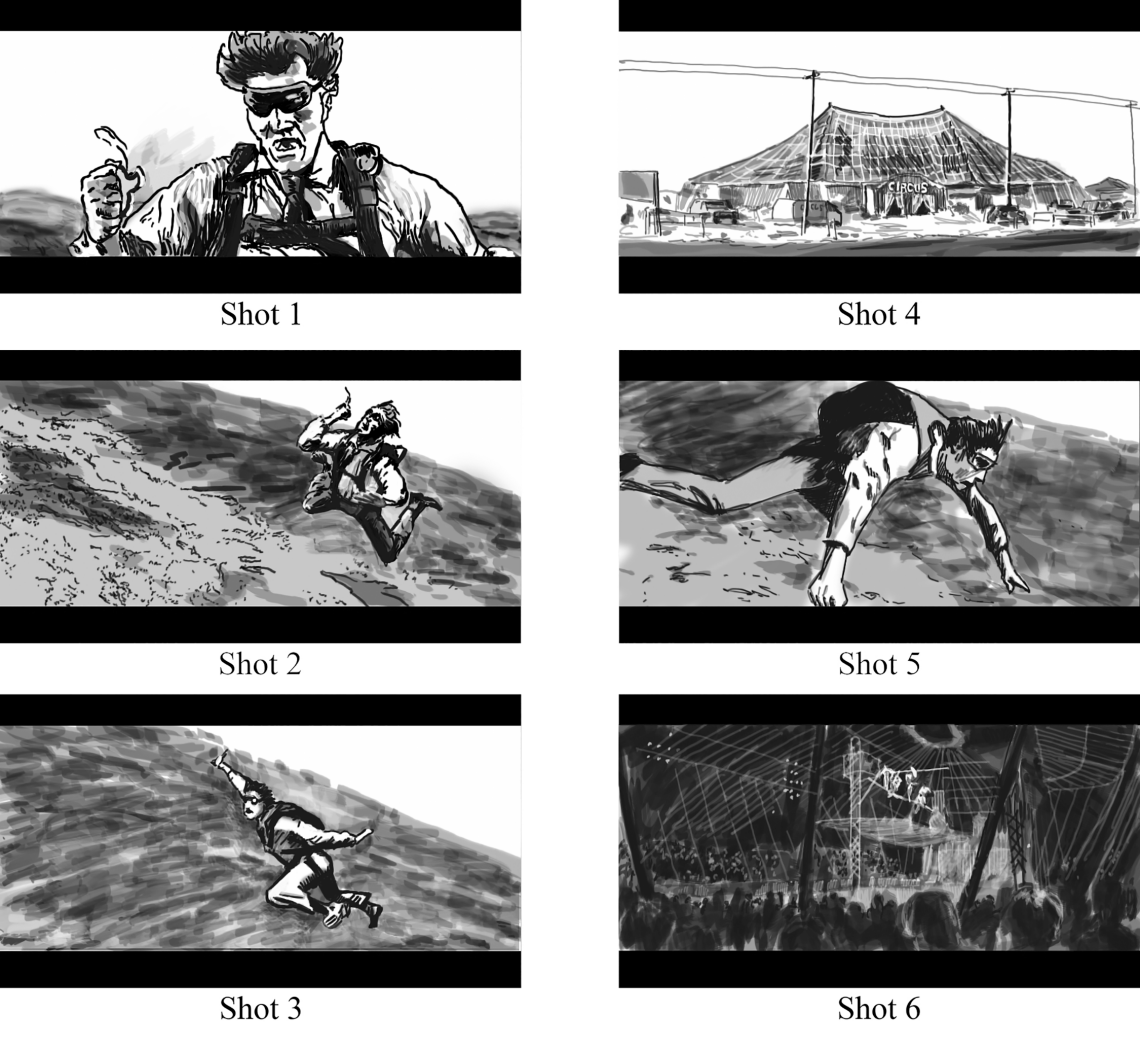
Illustrative frames from the six shots in the 12 second clip of the film “Moonraker” [42].

Interestingly, narrative films generally seem not to take much cognitive effort to comprehend, which is amazing if one thinks about the structural features of film. Films are comprised of thousands of individual camera shots that are edited in such a way that they create an illusion of real events unfolding in a narrative world. This trick is accomplished by following highly conventional editing practices that likely facilitate viewers’ ability to map semantic features (e.g., objects, actions, goals) and movement across camera shots [41]. Consider the six shot sequence from the James Bond movie *Moonraker* [42] illustrated in Fig 1 (see also http://www.youtube.com/watch?v=fAucs8K5E0U). In approximately 12 seconds of the film it conveys a richly situated narrative event. In it, we see Jaws, an antagonist in the film, pull a ripcord of his parachute (Shot 1), it fails (Shots 1–3), and then we see a *cross-cutting sequence* of Jaws falling and of a circus tent (Shots 4–6). *Cross-cutting* is an editing convention that alternates shots between two different locations in the narrative world (here, Jaws falling through the air, and the circus tent). This convention usually engenders a predictive inference that the events taking place in the two locations will converge (e.g., the person falling through the air will fall on the circus tent), which nearly all viewers who watch this segment generate by the final shot, Shot 6 [36]. But that predictive inference depends upon viewers first constructing a mental model of the events, including establishing temporal and spatial relationships between the two events of Jaws falling and the circus, and to do so based on a rapidly changing visual stream. While the use of cross-cutting helps signal spatiotemporal relationships across shots [13, 43], viewers must coordinate their eye movements to extract the most important information from the visual stream in constructing their mental model of the film. This brings us back to our central question: what is the relationship between viewers’ eye movements while watching a film and their comprehension of it?

One possibility is that movie viewers’ mental models drive their eye movements. For example, recent eye movement studies have shown effects on viewers’ eye movements of perspective taking (imagining one is a home buyer or burglar while looking at home photos [44]) viewing task (‘spot the location’ vs. free-viewing videos [12]) and viewer expertise (tennis playing experience while watching tennis videos [45]). These findings confirm that endogenous higher-order cognitive factors can influence gaze in unedited film sequences (but see [46]).

However, given the finding of strong attentional synchrony when viewers watch highly edited films, a critically important question is whether such endogenous control of eye movements may override endogenous effects due to comprehension differences. Put differently, if film viewers are all looking at the same things at the same time (due to Hollywood film-making techniques), can their eye movements differ based on their individual mental models?

## Overview of the Study

The current study investigated the extent to which mental models can affect eye movements in the context of a highly composed film segment. We showed viewers the clip from *Moonraker* depicted in Fig 1, which was ideal for our purposes for several reasons. The segment is rapidly edited, usually having a single object framed at screen center, which should produce strong attentional synchrony. Furthermore, we know from prior research [36] that nearly all viewers of this segment generate the predictive inference, “[Jaws] will fall on the tent,” which requires them to establish temporal, spatial, and causal coherence across the shots in the cross-cutting sequence as they develop their mental model for the scene [13, 14, 36, 47].

Our method used what we call the “*jumped-in-the-middle*” paradigm, which derives from the common television viewer experience of turning on the TV in the middle of a film and trying to make sense of it. After some time, we come to understand the on-going narrative, but not at the beginning. Thus, the jumped-in-the-middle paradigm manipulates whether viewers have already developed a mental model of the narrative before watching the target film sequence. Thus, we thus compared two viewing conditions: those who saw only the 12 second clip, the *No-context condition*, and those who also saw the preceding 2 minutes and 56 seconds of the film, the *Context condition*, which showed an altercation between Bond and his enemies on a small jet plane and ended with Bond and Jaws being thrown out of the plane. The critical segment started just after Bond had pulled his ripcord and escaped from Jaws.

The study consisted of three experiments, each of which measured different dimensions of film viewers’ comprehension and perception of this clip. Experiments 1 and 2 tested whether participants in the Context condition had better comprehension of the clip than those in the No-context condition (although without having any more information about the circus tent), and specifically whether they were more likely to draw the critical inference of Jaws falling on the circus tent. We predicted this would occur because viewers in the Context condition would have already established a mental model of the situation, including the protagonist Jaws, his spatiotemporal context of free-falling, and his likely goal of using his parachute to land safely, prior to seeing the critical six shots. Having already established such a mental model, the failure of Jaws’ parachute would quickly be understood by viewers in the Context condition as an impediment to Jaws’ goal of using it to land safely. That, in turn, would make the potential relevance of the circus tent to stopping Jaws freefall more salient, thus facilitating generation of the critical inference. Experiment 3 then tested whether such possible differences in comprehension would be associated with differences in participants’ eye movements, or whether participants’ attentional synchrony would override any potential differences in eye movements due to differences in comprehension. More detailed predictions regarding differences between conditions are provided in the introduction of each experiment.

## Experiment 1: Think-Aloud

Experiment 1 used a think-aloud method consistent with Magliano et al. [36] to determine whether viewers interpret the shot sequence differently across the two viewing conditions. While participants produced verbal protocols after every shot in the clip, Shots 4–6 were most critical for the analyses of the think aloud protocols because they contained the cross-cutting sequence where the critical predictive inference should be generated [36]. We predicted that viewers in the Context condition would more appropriately interpret intent of the cross-cutting sequence and therefore have a higher likelihood of generating the predictions that the character will land on the circus tent than those in the No-context condition. We predict this would occur because viewers in the Context condition would have already established a mental model of the situation, including the protagonist Jaws, his spatiotemporal context of free-falling, and his likely goal of using his parachute to land safely, prior to seeing the critical six shots. Having already established such a mental model, the failure of Jaws’ parachute would quickly be understood by viewers in the Context condition as an impediment to Jaws’ goal of using it to land safely. That, in turn, would make the potential relevance of the circus tent to stopping Jaws freefall more salient, thus facilitating generation of the critical inference.

### Method

#### Participants

Ninety-eight participants were randomly assigned to either the Context or No-context conditions in equal numbers (49 per condition). All participants had 20/30 or better corrected or uncorrected vision. The Kansas State University Institutional Review Board approved the study, and determined that the study posed minimal risk to the participants, and informed consent was deemed unnecessary (i.e., exempt under the criteria set forth in the Federal Policy for the Protection of Human Subjects.) All participants received course credit for their participation, and all analyses were performed on anonymized data.

#### Stimuli

We used two clips from the James Bond film *Moonraker* [42], one lasting 3:08 (2:56 + 12 seconds) in the Context condition, and the other lasting 12 seconds in the No-context condition. To focus entirely on the use of visual information, the clips were presented silently (the sequence contained no critical dialogue or sound effects). Both videos had a frame rate of 30 fps, and a video resolution of 720 x 480 pixels. Each participant viewed the film on a 17” Samsung SyncMaster 957 MBS monitors at a fixed distance of 53.3 cm using a chinrest, with the screen subtending 27.06° x 18.18° of visual angle, and the monitor set to 60 Hz.

#### Procedures

Prior to viewing the film, participants were instructed that the film would stop at certain points. During the target 12 second film clip, immediately following each shot, the video clip disappeared and was replaced by a text box, which prompted the participant to report whatever thoughts immediately came to mind regarding what they thought was happening at the points when the film clip was stopped. The think-aloud protocols were produced by typing rather than speaking, but both types of verbal protocols have been shown to produce similar content [48]. When finished reporting, they pressed a button to go to the next shot. Participants in the Context condition were also probed for their thoughts for the three shots preceding the critical 12 second clip in order to allow them to “off-load” a general description of what had happened over the previous 2 minutes and 56 seconds minutes and thus only report contents presented during the target 12 second clip. After watching the complete clip, all participants were asked if they had previously seen the film, and if so, to type its title. Data from 10 participants who correctly reported “Moonraker” or a James Bond film was discarded.

#### Protocol analysis

The think aloud protocols for the cross-cutting sequence (i.e., Shots 4–6) were of particular interest because it is during this sub-sequence that the critical inference (i.e., [Jaws] will land on the tent) should be generated [36]. The analysis of the think aloud protocols targeted two idea units that were important for revealing differences in how the cross cutting-sequence was understood across two viewing conditions: “X will land on the tent” and “there is a tent/building/structure” (Cohens Kappa were .96 and .91, respectively). Participants were only given credit for the first instance of mentioning these idea units.

### Results and discussion

**Fig 2:**
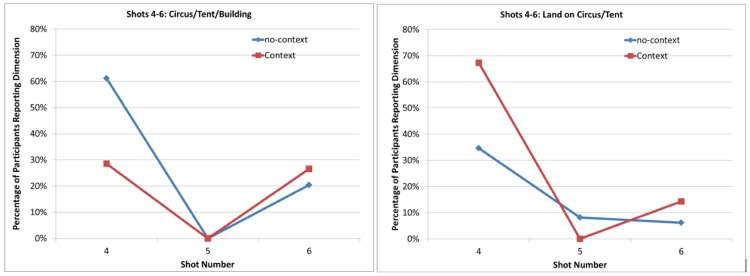
Each graph represents the proportion of participants who reported a critical idea over Shots 4–6 in the Context and No-context viewing conditions. Circus/Tent/Building = mention the circus tent or other building; Land on Circus/Tent = mention of landing on the circus or building.

Table 1 shows the frequency data, which were analyzed using a 2 (viewing condition [Context vs. No-context]) x 2 (critical idea [reported vs. unreported]) contingency table for both critical thoughts. Data from the think-aloud protocols are presented in Fig 2 for Shots 4–6, showing the proportion of participants who reported each critical idea as a function of the shot.

**Table 1:** Frequency of participants that reported the critical thought between the Context and No-context viewing conditions.

	Land on Tent		There is a Circus Tent	
Critical Thought	Reported	Not Reported	Reported	Not Reported
Context	40	9	26	23
No context	24	25	40	9

As shown in Fig 2, the results for Shots 4–6 were consistent with our predictions. Most importantly, the viewing condition affected reporting that “[Jaws] will land on the circus tent,” (*χ* ^*2*^ (1) = 11.53, *p* = .001)(Fig 2, right panel). Across Shots 4–6, the Context condition reported the critical inference more often than the No-context condition. Conversely, the opposite effect can be seen for the more general information describing the “circus tent” (*χ* ^*2*^ (1) = 9.09, *p* = .003)(Fig 2, left panel). Specifically, after Shot 4, viewers in the No-context group more frequently reported the more general information (e.g., “there is a tent”) than those in the Context condition, though this difference disappeared in Shot 6, the second appearance of the circus tent. These results clearly show differences in viewers’ understanding based on their mental models of the narrative. Namely, without context, viewers had difficulty interpreting the communicative intent of the cross-cutting, which required them to bridge the apparent lack of coherence between the different scenes (i.e., Jaws in the air, the circus on the ground). However, with an appropriate mental model, the critical predictive inference could resolve this coherence gap [49].

## Experiment 2: Event Segmentation

Experiment 2 investigated the process of event segmentation as the movie clips were viewed. Event segmentation is an important aspect of event model and mental model construction [13, 47, 50–52]. There is strong evidence that segmentation is an obligatory and spontaneous part of event understanding [51, 53], and *event segmentation tasks* have frequently been used to investigate event perception in both naturalistic and narrative films [14, 52, 54]. In this task, participants identify perceived changes in the depicted events, usually by pressing a button, which generally show good inter-subject and test-retest reliability.

In narrative films, viewers tend to perceive new events when there are shifts in situational continuities, such as time, space, and causality (e.g., [13, 55]). However, there is evidence that the cinematic device of cross-cutting can reduce the likelihood of viewers perceiving event boundaries when there is an abrupt change in location, such as from Shot 3 to Shot 4 [13, 43]. Specifically, viewers may assume that the shots of the new location (e.g., Shot 4) are relevant the unfolding scene [56] and thus be less likely to perceive it as a new event. Thus, we predicted that the No-context condition would be more likely to identify new events during Shots 4–6 than the Context condition, providing further evidence of differences in comprehension between the two viewing conditions.

### Method

#### Participants

88 participants were randomly assigned to either the Context or No-context conditions (44 each). All participants had 20/30 or better corrected or uncorrected vision. All ethical considerations were the same as Experiment 1.

#### Stimuli

The stimuli were identical to those in Experiment 1.

#### Procedures

Before viewing the *Moonraker* video clip, participants practiced an event segmentation task using an 83 second silent video of a person doing the laundry. After segmenting the *Moonraker* clip, participants were asked if they had previously seen the film, and if so, to type the title. Data from five participants were discarded due to having seen the film.

### Results and discussion

**Fig 3:**
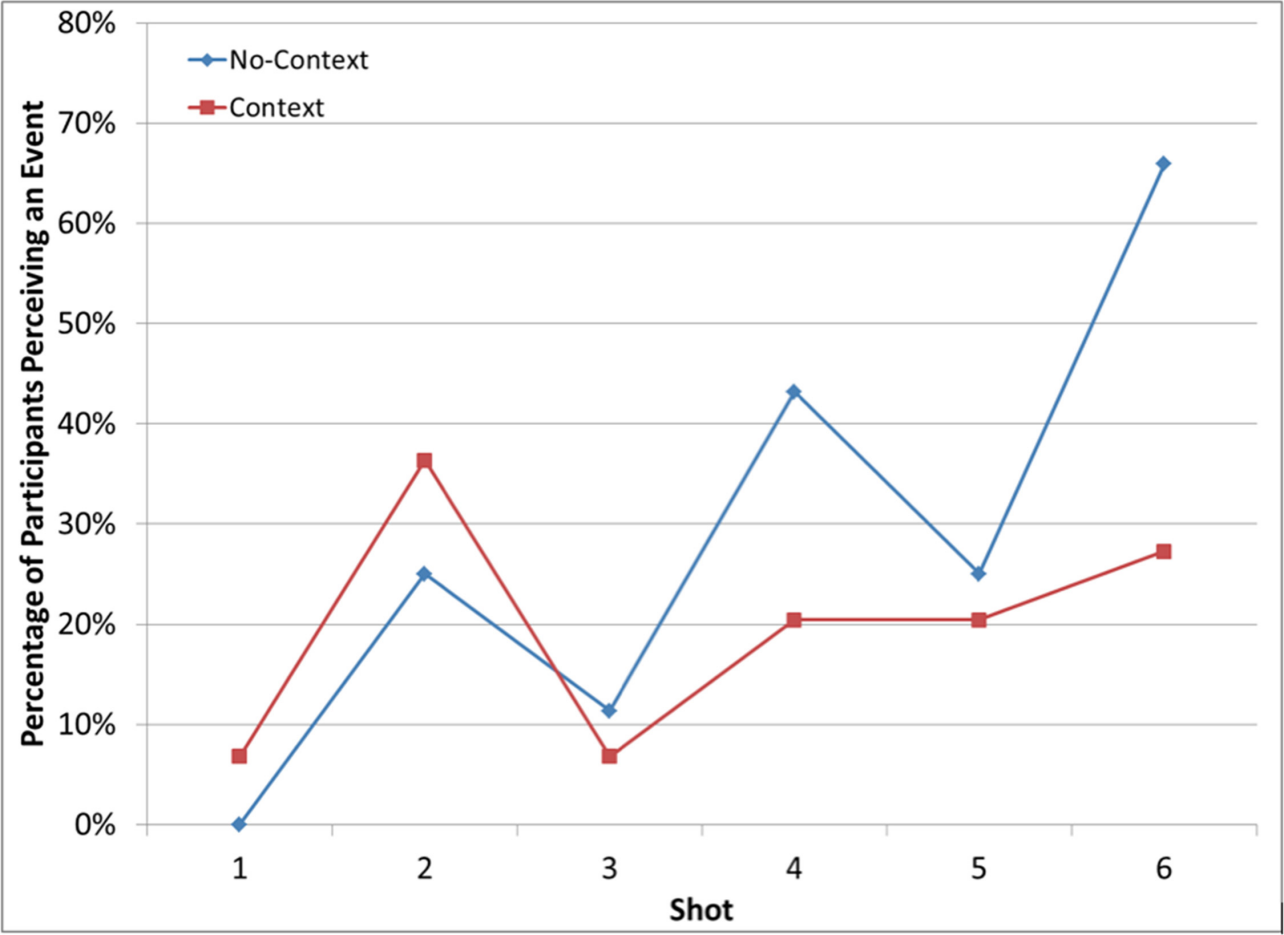
Percentage of participants who perceived new events in Shots 1–6 in the Context and No-context viewing conditions.

Fig 3 shows the percentage of perceived events for both the Context and No-context conditions as a function of the shot number. Table 2 shows a 2 (viewing condition [Context vs. No-context]) x 6 (Shot [1–6]) contingency table for frequency of events perceived. Overall, this analysis indicated little difference in the frequency of perceived events over the course of the six shots, χ2 (5) = 9.79, *p* = .08. A chi-square test of independence showed no difference between conditions in Shot 2, χ2 (1) = 0.36, *p* = .55, and no chi-square could be computed for Shots 1 and 3 due to having five or fewer observations in some cells, though they are not visually different. (The increase in event segmentation for both Context and No-context conditions in Shot 2 was likely due to viewers’ perception of Jaws’ ripcord breaking in that shot, as shown in think-aloud protocols for Shot 2. We have not reported the think-aloud results for Shots 1–3 because they are less central to the research questions investigated in this study.) However, Fig 3 shows that the cross cutting in Shots 4–6 did produce differences between the viewing conditions, with the No-context condition more likely to perceive Shots 4 (χ2 (1) = 4.17, *p* = .04) and 6 (χ2 (1) = 5.77, *p* = .02) as new events than the Context condition. No difference was observed for Shot 5, χ2 (1) = 0.89, *p* = .35. Thus, viewers in both conditions similarly interpreted the event structure of Shots 1–3, but differed in how they interpreted the event structure of the cross-cutting sequence in Shots 4–6. As predicted, the No-context condition was more than twice as likely as the Context condition to perceive new events at the introduction of the new spatial location, indicating that No-context condition perceived less coherence in the cross-cutting sequence (i.e., they had less understanding of the sequence).

**Table 2:** Frequency of events perceived for each shot in the Context and No-context conditions.

	Shot Number					
Viewing condition	1	2	3	4	5	6
Context	3	14	3	7	7	12
No-context	0	11	5	17	11	27

## Experiment 3: Eye Movements

Experiment 3 measured viewers’ eye movements while they watched the 12 second movie clip, and at the end, we asked them to predict what would happen next. The critical question was whether the differences observed in comprehension between the Context and No-context conditions in Experiments 1 and 2 would be evident in the eye movement data.

We used eye movements to measure two functional aspects of film cognition, attentional synchrony, and processing load. To quantify attentional synchrony, we developed a new measure, Z-normalized gaze similarity, which allowed us to compare the similarity of each viewer’s gaze location, at a given point in time, to the distribution of gaze positions of all other viewers in the same viewing condition, and between conditions. This metric modifies the Normalized Scanpath Similarity metric [24] but preserves individual participants in the analysis, allowing one to perform inferential statistics. To quantify processing load, we measured the probability of fixation, which varies monotonically with fixation duration, a standard measure of processing load, but which can be binned more easily than fixation durations. Both measures are justified and described in greater detail in the “Results” section.

It is quite possible that the intensified continuity style of filmmaking [29], such as the use of many brief shots as in the current study, creates such a high level of attentional synchrony that there may be no differences across the two viewing conditions, which we call the *tyranny of film* hypothesis. This hypothesis is supported by a number of recent studies showing high attentional synchrony across viewers while watching Hollywood style films, [9, 24, 25, 37, 57, 58] including similarities between monkeys and humans watching the same film clips [59, 60], and between people watching scrambled and unscrambled versions of the same film clips [26].

Conversely, it is possible that the clear differences in viewers’ comprehension while watching the clip, shown in Experiments 1 and 2, will create differences in attentional synchrony between the two viewing conditions. Such differences should be strongest in Shot 4, in which the No-context condition viewers were more likely to perceive a new event (Exp. 2), and the Context condition viewers were more likely to draw the critical inference (Exp. 1). We therefore call this the *mental model* hypothesis, which could go in either of two directions. The viewers in the Context condition, who have a more coherent mental model of the narrative, could show greater attentional synchrony because they would be more likely to pay attention to the same important information. Conversely, viewers in the No-context condition could be more likely to attend to visually salient imagery due to their lack of a mental model and thus exhibit more attentional synchrony, because they would be paying attention to the same visually salient imagery. Either prediction seems valid.

Regarding processing load, as measured by probability of fixation, the tyranny of film hypothesis would again suggest the null hypothesis. If everyone is looking at roughly the same places at the roughly the same times, there should be little room for differences in processing load to be evidenced in eye movements. Conversely, the mental model hypothesis predicts that those in the No-context condition would have to work harder to build a mental model of the events in the film clip, since they did not start with one in memory, and thus would show evidence of greater processing load in their eye movements. Finally, the tyranny of film and mental model hypotheses may not be mutually exclusive, but could depend on the dependent measure under consideration. It is possible that we support the tyranny of film hypothesis with respect to attentional synchronicity, but the mental model hypothesis for processing load.

### Method

#### Participants

176 participants were randomly assigned to either the Context or No-context conditions in equal numbers (87 per condition). (Given that the eye movement data in the study came from a 12 second movie clip, there were on average only 31 fixations per participant. This small number of observations per participant necessitated collecting data from a large number of participants.) All participants had 20/30 or better corrected or uncorrected vision. All ethical considerations remained the same as in Experiments 1 and 2.

#### Stimuli

The stimuli were identical to those in Experiments 1 and 2, except that, in the current experiment, the movie was viewed on a 17” ViewSonic Graphics Series CRT monitor (Model G90fb) at a fixed distance of 61 cm, using a forehead and chinrest, with the screen subtending 21.42° x 16.10° of visual angle.

#### Procedures

Participants were simply told that they would be shown a video clip while their eyes were tracked. We used an EyeLink2000 eyetracker (2000 Hz sampling rate; average spatial accuracy = 0.5 degrees). After calibrating the participants (9-point), the procedure differed slightly between the Context and No-context conditions. Context condition participants saw a central fixation point, and while fixating it, pressed a button initiating the 3:08 film clip (unknown to participants, the last 12 seconds [0:12] of which was the critical 6-shot sequence). In the No-context condition, participants saw a central fixation point, and while fixating it, pressed a button. The fixation point then jumped to a position 13.65° to right of center. Once they fixated the new fixation point, they saw the fixation point jump back to the center. When they made a saccade (defined as a saccade velocity > 30° per second) toward the new central fixation point, the 12 second No-context film clip began. This ensured that the film would already be on the screen at the moment their eye movement ended and their first fixation began. This eliminated the possibility of a motion transient being created during viewers’ first fixation by the onset of the film, which would produce saccadic inhibition, thus lengthening their fixations [61, 62]. It is known that whenever a new shot begins in a film, viewers tend to make a saccade to the center of the screen [24]. Thus we expected that viewers in the Context condition would do so at the beginning of the first shot of the 12 second clip, and our procedures ensured that those in the No-context condition also did so. After the video, all participants then saw a screen that asked them “What will happen next?” and they used a keyboard to fill in a text box with their answer. They were then asked if they had seen the movie before, and if so, to type its title. Data from 13 participants who correctly reported that the movie clip came from “Moonraker” or a James Bond film was discarded.

### Results and discussion

#### Predictive inference

Asking viewers what they predicted would happen next allowed us to directly connect a given viewer’s understanding of the film sequence with their eye movements while they had watched it. We coded participants’ responses for mention of falling on the circus tent. Consistent with Experiment 1, significantly more viewers in the Context condition, (91%) made the critical inference than in the No-context condition (71%), χ2 (1) = 10.81, *p* = .001.

Only the participants who drew the critical inference in the Context condition were entered in subsequent analyses (referred to as ‘Context + Inference’). There were too few Context participants who failed to draw the critical inference (n = 8) for further analysis. The No-context participants were analyzed as those who did or did not draw the inference (hereafter, *Inference* vs. *No-Inference*).

#### Attentional synchrony

To compare attentional synchrony between viewing conditions, we needed a measure of the spatiotemporal distribution of gaze. We modified a common technique for measuring gaze clustering during film viewing—treating gaze as a two-dimensional probability distribution (used in Normalized Scanpath Salience, Gaussian Mixture Modelling, and Kullback Leibler Divergence; for review see [63])—but retained individuals in the analysis. Our metric, which we call *gaze similarity*, is an adaptation of the Normalized Scanpath Saliency (NSS) first proposed by Peters et al. [64] and extended to video by Dorr et al. (for details of the method and equations, see [24]).

We have modified this method in two critical ways. First, to calculate inter-observer similarity, a probability map is created by plotting 2D circular Gaussians (1.2° SD ≈ the fovea) around the gaze locations within a specific time window (225 ms ≈ an average fixation [24]) for all but one participant within a condition. These Gaussians are summed and normalized relative to the mean and SD of these values across the entire video (z-score similarity = (raw values–mean) / SD). The gaze location of the remaining participant is then sampled from this distribution (i.e., a Z-score is calculated for this participant) to identify how their gaze fits within the distribution at that moment. This *leave-one-*out procedure is repeated for all participants within a condition until each participant has a z-scored value (referred to as *gaze similarity* here). These values express both 1) how each individual gaze location fits within the group at that moment and 2) how the average gaze similarity across all participants at that moment differs from other times in the video: A z-score close to zero indicates average synchrony, negative values indicate less synchrony than the mean (i.e., more variance), and positive values indicate more synchrony.

**Fig 4:**
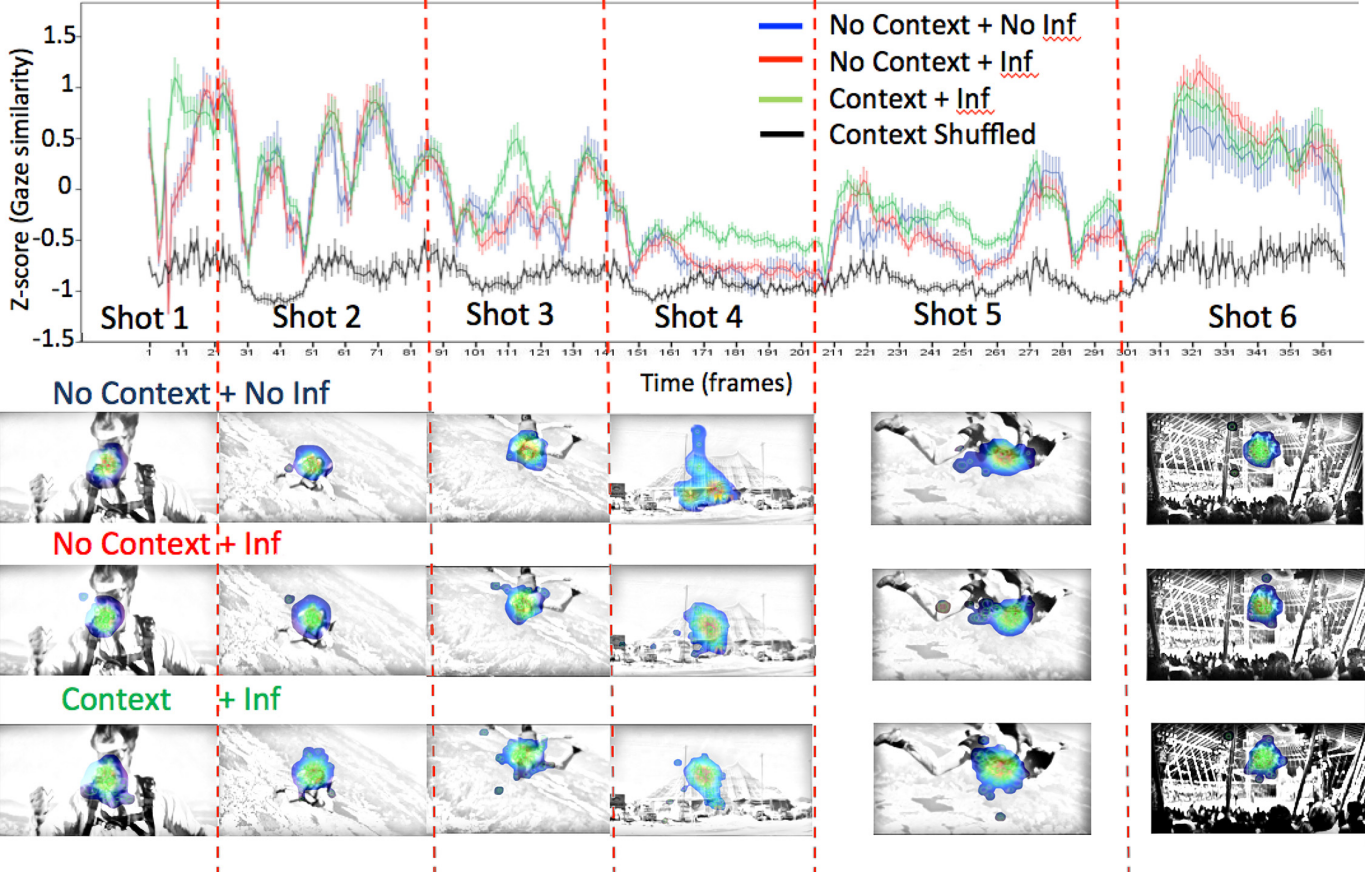
Similarity in gaze distribution as a function of viewing condition + inference group (No-context + No-inference [blue], No-context + Inference [red] and Context + Inference [green], Shuffled Baseline [black] conditions) and Shot (1–6). Gaze similarity is calculated relative to the Context + Inference group. Similarity is expressed as z-scored probabilities relative to the mean Context + Inference group gaze probability distribution. Values below zero indicate less attentional synchrony than the mean for the Context + Inference group; values above zero indicate higher attentional synchrony than the mean. Error bars indicate +/- 1 standard error across individuals for each frame.

**Fig 5:**
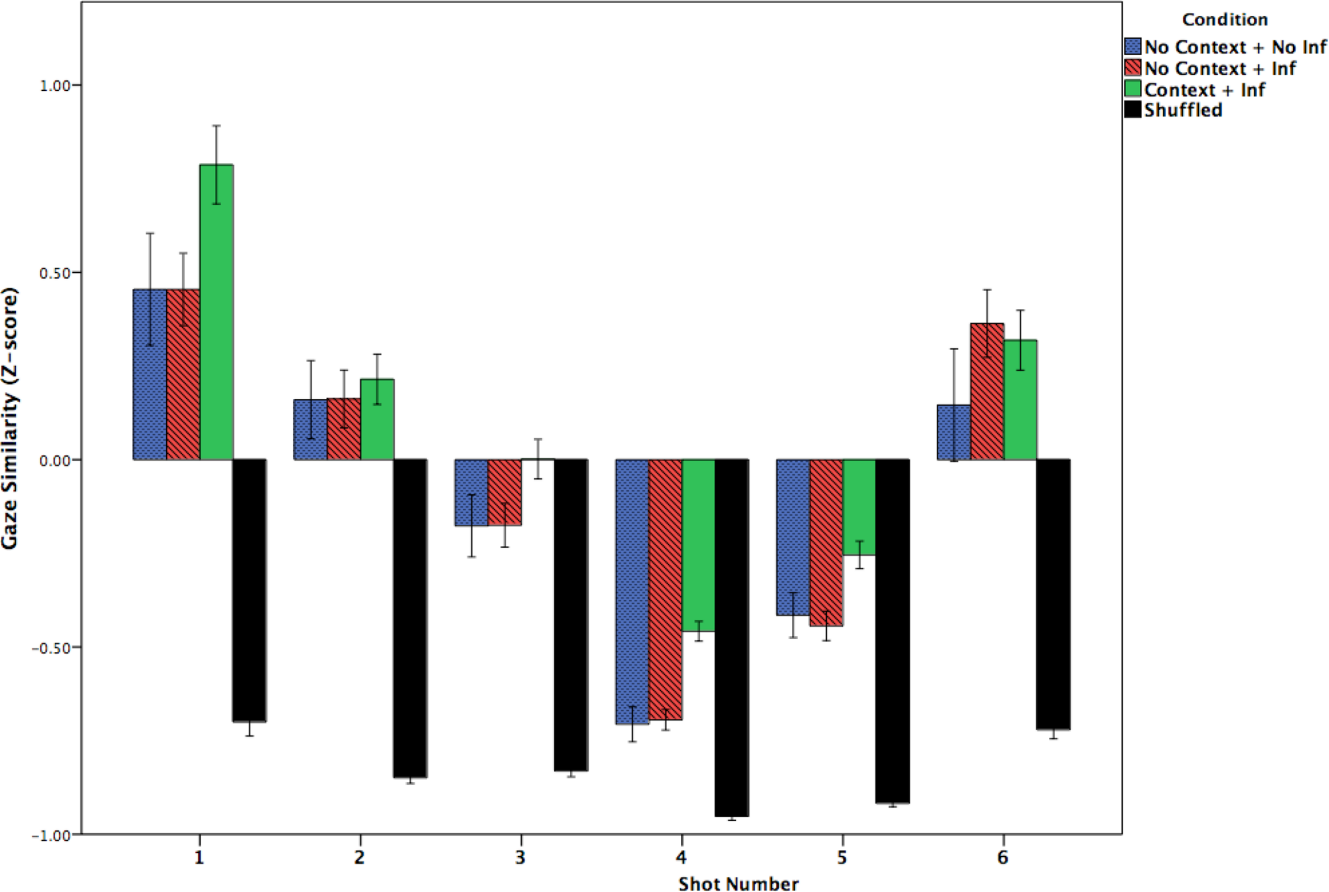
Similarity in gaze distribution as a function of the viewing condition + inference group (the No-context + No-inference [blue], No-context + Inference [red] and Context + Inference [green] conditions) and Shot (1–6). Similarity is expressed as z-scored probabilities relative to the Context + Inference baseline gaze probability distribution. Larger values indicate greater attentional synchrony, while lower values indicate less attentional synchrony.

Second, the method is extended to allow gaze from different viewing conditions (e.g., No Context + No Inference; referred to as condition A in this example) to be sampled from a reference distribution (Context + Inference; referred to as condition B in this example). For each gaze point in condition A, the probability that it belongs to condition B is identified by sampling the value at that location from B’s probability distribution (this time *leave-one-out* is not used as the gaze does not belong to the same distribution so cannot be sampled twice). The resulting raw NSS values for condition A are then normalized to the reference condition, B. Importantly, if the two distributions are identical, the average z-scored similarity for distribution A will fluctuate identically to reference distribution B, expressing more (positive z-score) or less (negative z-score) attentional synchrony over time (see Figs 4 and 5). However, as the similarity score is derived from the reference distribution, B (i.e. Context + Inference), if the two distributions differ significantly we cannot know if this is because the comparison distribution (i.e., No Context + No Inference) has more or less attentional synchrony than the reference distribution. We can only say that the comparison distribution differs more from the reference distribution than the reference distribution differs from itself. For example, both gaze distributions could be tightly clustered but in different, non-overlapping parts of the screen or the comparison distribution could be more spread out and only partly overlap with the tight reference distribution. Both situations would result in significant differences between the distributions.

Also, in order to identify if attentional synchrony is significantly greater than would be predicted by chance, a shuffled baseline condition is created. The shuffled condition is derived from the reference distribution (i.e. Context + Inference) but the relationship between gaze location and time (frame number) is eradicated by shuffling the order of gaze samples across all frames within a particular participant. This creates a new set of gaze locations for each frame that are derived from the range of locations fixated throughout the video by a particular participant, but not necessarily at that particular moment. Thus, the shuffled baseline represents a generous measure of the minimum degree of attentional synchrony that would be created by random (see Mital et al., 2010; for a similar approach).

Since a mean gaze similarity is calculated for each participant, we can therefore statistically compare these values to identify moments when either 1) two conditions significantly differ from each other, or 2) when a condition has greater attentional synchrony than would be predicted by chance (i.e. the shuffled baseline). This modification of the metric allows variation across individuals within and between groups to be statistically compared. This is statistically more robust than comparing differences across videos or arbitrary chunks of a video as is typically done using the traditional form of NSS [12, 24].

#### Attentional synchrony results

To get a first impression of the gaze behavior across the different viewing conditions, dynamic heat-maps of the two videos can be qualitatively analyzed (Video 1; heat-maps represent a 2° radius Gaussian plotted around each raw gaze point for that frame, summed and normalized: http://www.youtube.com/watch?v=-4-aKvOpPvI). Stills of these heat-maps are shown in Fig 4. Examining the dynamic heat-maps across the three participant groups suggests that gaze behavior was very similar, supporting the tyranny of film hypothesis. However, such analyses may be too coarse to capture the micro-dynamics of gaze. A more robust method for comparing gaze distributions is to quantify the distribution of gaze at each moment using the *gaze similarity* metric.

Although the tyranny of film hypothesis predicted that gaze similarity would be indistinguishable across viewing conditions due to filmmaker control, this prediction was not statistically supported. Specifically, a 3 (Participant Group: No-context + No-inference vs. No-context + Inference vs. Context + Inference) by 6 (Shot number: 1–6) mixed ANOVA, revealed a significant effect of participant group, *F*(2, 163) = 3.978, *p* = .021, *pη2* = .047, a significant within-subject effect of shot number, *F*(5, 815) = 122.9, *p* = .001, *pη2* = .430, and a non-significant trend towards an interaction between the two, *F*(10, 815) = 1.70, *p* = .076, n.s., *pη2* = .02. To explore these viewing condition effects within each shot, we performed Bonferroni corrected post-hoc t-tests. Across all shots, the mean gaze similarity in the Context + Inference group was nearly but non-significantly higher (i.e., more attentional synchrony; *Mean* = 0.102, *SD* = .382) than in the No-context + Inference group (*Mean* = -0.056, *SD* = .383, *p* = .054, Cohen’s *d* = 0.42) and a weaker non-significant trend to be higher than the No-context + No-inference group (Mean = -0.09, SD = .388, *p* = .089, n.s., Cohen’s *d* = 0.505), most likely due to the smaller number of participants in this group. These comparisons show a non-significant trend for the gaze of each participant from the No-context groups was to be less similar to the Context + Inference group than the Context + Inference group was to itself. Much more clearly, all conditions had significantly greater gaze similarity than the shuffled baseline (all *p*s < .001, all Cohen’s *d*s > 2.986), indicating that all conditions exhibited a high degree of attentional synchrony. A comparison of effect sizes suggests that the *Tyranny of Film* effect (i.e., greater attentional synchrony than the shuffled baseline) is at least six times larger than the *Mental Model* effect (i.e., the difference between Context and No Context conditions).

The significant main effect of shot number indicates that the gaze similarities varied across shot number, as can clearly be seen in Fig 5. Across the three groups, attentional synchrony was highest in Shots 1 and 6 and lowest in Shots 4 and 5. Although the interaction between participant group and shot number did not reach statistical significance, inspection of Fig 5 suggests that the difference between the three experimental groups in Shots 1, 3, 4 and 5 appears greater than in the Shots 2 and 6. Group differences were confirmed by within-shot ANOVAs for shots 1, 3, 4, and 5 (all *p*s < .05) but not shots 2 and 6 (*F*s < 1). In Shot 1, this difference, in which the Context + Inference group showed greater attentional synchrony than the other No-context groups, was probably due to the gaze-contingent starting location of the No-context conditions or other subtle artifacts. Thus, we refrain from drawing any conclusions about Shot 1 and only include it in Fig 5 for completeness of data presentation. In Shot 3, the difference between the Context + Inference and the No-context + Inference groups showed a non-significant trend (*p* = .076, n.s., Cohen’s *d* = 0.38). Importantly, however, in Shot 4 where the circus tent is introduced, the difference between the Context + Inference and both No-context groups was larger than in any other shot (~-0.24) as both No-context groups had significantly lower gaze similarity scores than the Context + Inference group (No Context + Inference: *p* < .001, Cohen’s *d* = 1.045; No Context + No Inference: *p* < .001, Cohen’s *d* = 0.917), due to differing degree of exploration of the screen in the No-context groups. However, gaze in these No Context conditions was still significantly more clustered during Shot 4 than the shuffled baseline (No Context + Inference: *p* < .001, Cohen’s *d* = 1.65; No Context + No Inference: *p* < .001, Cohen’s *d* = 1.49; see Fig 5). Strikingly, a comparison of effect sizes shows that even in Shot 4, which had the lowest attentional synchrony, the *Tyranny of film* effect (i.e., greater attentional synchrony in the No-context groups than the shuffled baseline) was roughly 1.5 times larger than the *Mental Model* effect (i.e., greater attentional synchrony in the Context than the No-context groups).

The above pattern of results was also found in Shot 5, but less so than in Shot 4, with both No-context groups showing lower gaze similarity than the Context + Inference group, though only significantly so for the No-Context + Inference group (No-Context + Inference = -.44, SD = .31, *p* = .002, Cohen’s *d* = 0.602; No-Context + No-inference = -.42, SD = .305, *p* = .077, n.s., Cohen’s *d* = 0.51). In general, consistent with the endogenous control version of the mental model hypothesis, this analysis shows that when gaze similarity differed, it was more coordinated in the Context + Inference group, while gaze in both No-context groups was more exploratory, particularly during Shots 4 and 5 when mental model coherence was most challenged due to the introduction of cross-cutting. However, there were no differences between participants in No-context condition who drew the critical inference versus those who did not, and all groups remained significantly higher than the shuffled baseline, again showing strong support for the *Tyranny of Film* hypothesis.

Fig 4 shows the fluctuating pattern of attentional synchrony over the course of the clip in more detail by expressing the gaze similarity scores for each video frame across the three viewing condition + inference groups. The now familiar pattern of gaze clustering immediately following cuts [24, 25] can be clearly seen here: 1) immediately following a cut, gaze was scattered as participants made eye movements at different times to the new center of interest (creating a dip in gaze similarity measure down to the level of the shuffled baseline), 2) then, about ten frames after each cut (~333 ms), gaze similarity increased as all participants, irrespective of viewing condition + inference group, looked towards the screen center. Following this initial rise in attentional synchrony following a cut, attentional synchrony decreased over the course of each shot as viewers began exploring different parts of the screen and gaze similarity decreased. Peaks in gaze similarity only occur within a shot when a center of interest suddenly moves, such as Jaws’ face in Shots 2, 3, and 5, or the trapeze artist in Shot 6. During these movements, the gaze distribution was very similar across all three viewing condition + inference groups. Only during Shots 3, 4, and 5 were there any noticeable and interpretable differences between the three groups.

Of specific interest are the gaze similarity differences between viewing conditions in Shot 4, when the circus tent is first introduced. There, viewers in both No-context groups showed less gaze similarity than the Context + Inference group with their gaze similarity dropping to the same level as the shuffled baseline about 20 frames into the shot. This suggests greater exploration of the incongruous shot by viewers having an impoverished mental model for the film clip. Greater exploration of the shot by the No-context participants also occurred at key moments during Shots 3 and 5 (both shots of Jaws struggling in freefall), suggesting that the absence of context can also lead to less attentional synchrony even in more active scenes. However, in the No-context condition, there was no difference in attentional synchrony between the participants who did or did not draw the critical inference across the entire film clip. This raises the question, why, during Shot 4 didn’t the No-context + Inference viewers show greater attentional synchrony like the Context viewers did? A possible explanation is that the No-context + Inference viewers did not make the critical inference during Shot 4, but did at some later point (e.g., during Shot 6, which showed the circus tent interior).

#### Processing load

When film viewers are so similar in where they look, eye movement measures that capture differences in processing time, like eye fixations durations, become critical. However, computing the mean fixation duration per shot is problematic. Specifically, when a fixation spans the boundary between two shots, assigning the fixation to either shot would be arbitrary. Likewise, dividing the fixation between the two shots would create artificial fixations with short durations. Thus, to avoid these problems, a new processing load measure was used—foveation probability. To calculate this measure, one determines whether a given viewer’s eyes were foveating for each frame in a shot. The number of foveated frames per shot are summed and divided by the number of frames in the shot. Thus, a participant who foveated 75 frames in a 100 frame shot would have a foveation probability of 0.75. Importantly, foveation probability is positively correlated with mean fixation duration. (For example, consider a 1,000 ms shot, in which Viewer A made two 450 ms fixations and two 50 ms saccades, and Viewer B made four 200 ms fixations and four 50 ms saccades. Viewer A would have a mean fixation duration of 450 ms and a foveation probability of .93, while Viewer B would have a mean fixation duration of 200 ms and a foveation probability of .87. Thus, as mean fixation duration increases, so too does the foveation probability.) Thus, we predict that as processing load increases, so too should the foveation probability.

**Fig 6:**
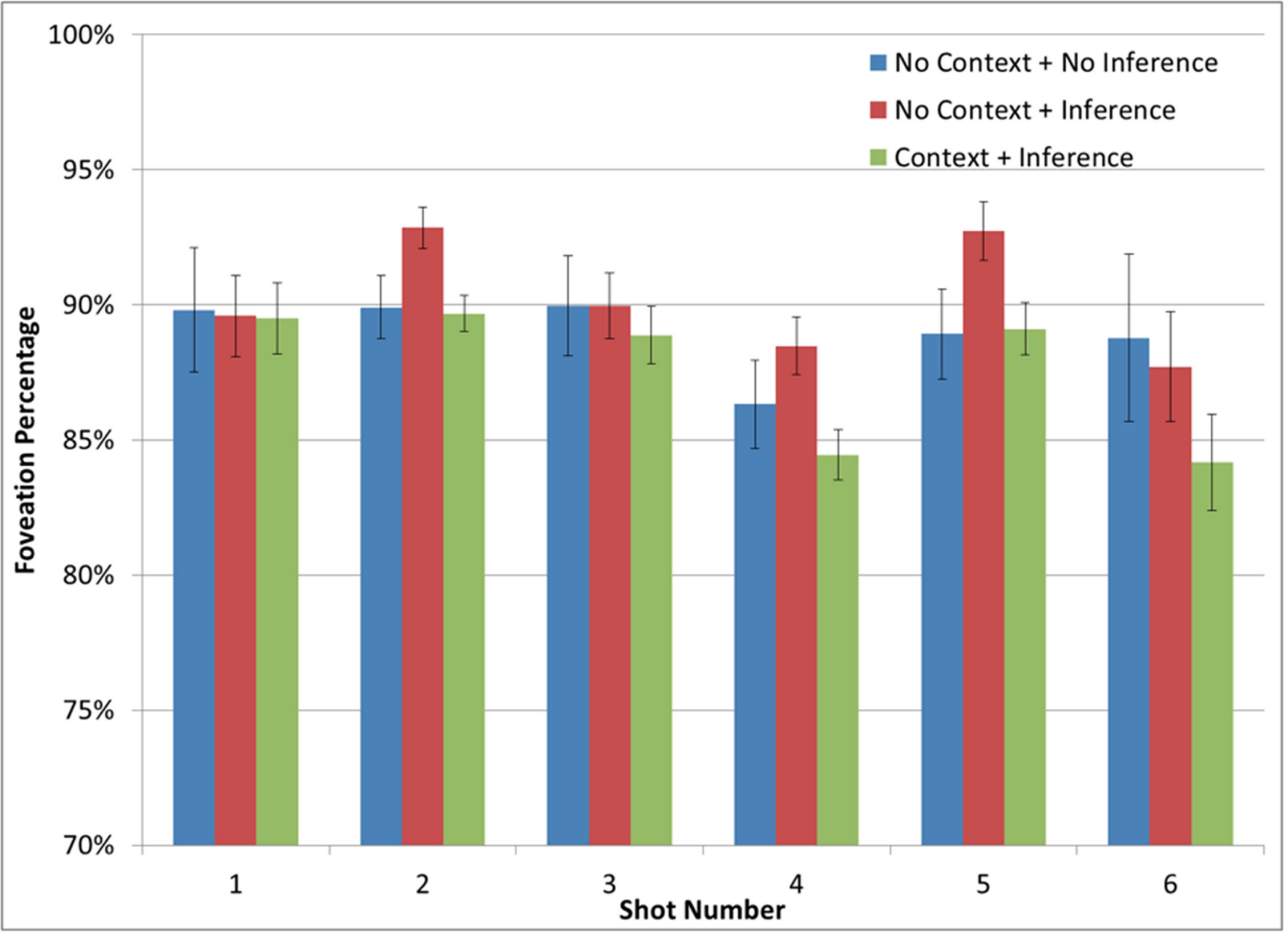
Mean foveation probability as a function of the viewing + inference condition (the No-context + No-inference [blue], No-context + Inference [red] and Context + Inference [green], Shuffled Baseline [black] conditions) and Shot (1–6). Error bars represent the standard error.

Foveation probabilities were entered into a 3 (Participant Group: No-context + No-inference vs. No-context + Inference vs. Context + Inference) by 6 (Shot number: 1–6) mixed ANOVA. As shown in Fig 6, there was a main effect of shot, *F* (5, 815) = 5.25, *p* < .001, *pη2* = .031, and a non-significant trend towards a main effect of Participant Group on foveation probability, *F* (2, 163) = 3.02, *p* = .051, *pη2* = .036. Specifically, those in the No-context + Inference group (*M* = 90.4%; *SD* = 4.5%) had greater foveation probabilities than those in the Context + Inference group (*M* = 87.4%; *SD* = 6.7%), *t* (138) = 3.01, *p* = .003, Cohen’s *d* = 0.53, but not those in the No-Context + No-inference group (*M* = 88.8%; *SD* = 7.9%), *t* = 1.20, *p* = .23, Cohen’s *d* = 0.25. These results provided mixed support for the Mental Model hypothesis. There was no interaction between shot and the Participant Group on foveation probability, *F* (10, 815) = 0.89, *p* = .54, *pη2* = .011. However, Fig 6 clearly shows this was not the case. The primary differences in foveation probability between those in the No-context + Inference group and those in the Context + Inference group were in Shots 2, 4, and 5, *t*s (138) ≥ 2.88, *p*s ≤ .005, Cohen’s *ds* ≥ 0.50. (The significant difference in Shot 2 was likely related to the increased event segmentation in Shot 2 (see Fig 3). This in turn was likely due to viewers’ perception of Jaws’ ripcord breaking in that shot, as shown in think-aloud protocols for Shot 2. We have not reported the think-aloud results for Shots 1–3 because they are less central to the research questions investigated in this study.) On the other hand, across all six shots there were no differences between the No-context + No-inference group and the Context + Inference group, *t*s (103) ≤ 1.21, *p*s ≥ .23, Cohen’s *d*s ≤ 0.29. Again, this latter result conflicts with the Mental Model hypothesis.

Perhaps the mixed support for the Mental Model hypothesis can be explained in terms of a further variable, such as effort after meaning [49, 65]. Specifically, for viewers having a depleted mental model (i.e., in the No-context condition), generating the critical predictive inference created a cognitive load (as shown by higher foveation probability for the No-context + Inference group than the Context + Inference group); however, this cognitive load could be avoided by simply not making the effort to generate the inference [66](as suggested by the lack of difference between the No-context + No-inference group and the Context + Inference group). On the other hand, for viewers having an adequate mental model of the narrative (i.e., in the Context condition), generating the predictive inference was less of a cognitive load, thus allowing them to make more eye movements during the shot. These results are clearest when viewers were faced with the cross-cutting sequence (Shots 4–6).

In summary, our analyses of attentional synchrony showed strong evidence that the filmmakers influenced when and where viewers attended, irrespective of their viewing condition or whether they made the critical inference. This was shown by the large and statistically significant differences between each of the viewing conditions and the shuffled baseline condition, which strongly supports the Tyranny of Film hypothesis. Nevertheless, there was also evidence of subtle influences of the viewer’s understanding on gaze behavior, with the context condition showing significantly greater gaze similarity and lower foveation probabilities, consistent with the mental model hypothesis. These differences were strongest in Shot 4, where Experiments 1 and 2 showed clear differences in understanding of the movie clip. Nevertheless, when we compared the effect sizes associated with the *Tyranny of Film hypothesis* (each viewing condition vs. the shuffled baseline) and the *Mental Model* hypothesis (e.g., the Context+Inference condition versus the two No-Context conditions), we found *Tyranny* effects 1.5–6 times larger than *Mental Model* effects. Thus, the most robust result was the overwhelming attentional synchrony created by the intensified continuity editing style used in the film.

## General Discussion

Our overarching research question in this study was, to what degree are movie viewers’ eye movements affected by their higher-level comprehension of a movie as they watch it? The answer to this question is especially interesting when compared to other task domains in which the eye movements and cognition relationship has previously been investigated, such as reading or static scene viewing. What makes this comparison novel and interesting is that highly produced films are known to produce strong attentional synchrony [24–26], likely due to filmmakers’ manipulation of motion, luminance contrast, image framing, and the short processing times afforded by short shot lengths (collectively called the intensified continuity style of filmmaking [29]). We call the combined force of these exogenous cues on viewers’ attention *the tyranny of film*, and it poses a stronger test of the eye movements and cognition relationship than static scene viewing (which lacks motion, or inherent limits on viewing time) or reading (in which viewing time is generally self-determined). Against such exogenous factors, we have pitted processes involved in higher-level narrative comprehension, specifically those involved in making a predictive inference, which are quintessentially *cognitive* in nature. Furthermore, for any viewer watching a movie, achieving such higher-level comprehension of it is arguably their implied task. Thus, the question of the degree to which higher-level comprehension processes influence viewers’ attention and eye movements while watching movies is theoretically meaty.

To study this question, we therefore chose a film clip from a James Bond movie using the intensified continuity style that was known to strongly engender a predictive inference at the end of the clip through the use of the cross-cutting editing technique [36]. We then manipulated viewers’ comprehension of the clip using what we call the “jumped in the middle” paradigm, which varied the amount of film context seen prior to watching the target film clip, while holding all aspects of the film stimulus constant. The results of three experiments showed that 1) having access to a mental model clearly facilitated comprehension of the film clip, consistent with the subjective experience of jumping into the middle of a movie, but 2) this was associated with only subtle measurable differences in eye movements, due to the overwhelming evidence of attentional synchrony. (It should be noted that the greater duration and content of the film clip seen by the Context condition, in addition to providing a narrative frame for the critical sequence, might also have subtly affected our outcome measures. For example, in Experiment 1, participants in the Context condition filled out three additional text boxes prior to the critical sequence. While this may have made the answers for the following textboxes more similar across viewing conditions, as intended, it may also have trained viewers of the Context condition to formulate their thoughts more appropriately. Also, in Experiment 2, watching a three-minute clip may have led Context condition viewers to apply more coarse-grained event segmentation than watching a 12 second clip.)

Nevertheless, a key concern for the current study is regarding the generalizability of the current results based on a single film clip. We have thus carried out a follow-up series of experiments using a different clip [67] from Orson Welles’ film “Touch of Evil” [68], which was a single long shot (i.e., no cuts) lasting 3 mins, 20 secs. We hypothesized that an extended single shot would be more likely to show differences in eye movements due to differences in comprehension, because attentional synchrony would not be artificially inflated by cuts (after which all viewers immediately fixate the center of the screen, as shown in Fig 4). We also used a stronger context manipulation. The Context condition viewers watched from the beginning of the clip, which shows a time bomb being put into the trunk of a car, after which the unwitting drivers of the car appear, get into the car, and slowly drive down the street. The No-context condition viewers were not shown the bomb. The clip continued for another 3 minutes, during which time the car is repeatedly seen, while two other characters walking on the street are introduced. The clip ended just before the full film shows the bomb explode, and we asked the viewers what they thought would happen next. As expected, the majority of Context viewers predicted that the car would explode, while essentially none of the No-context viewers did. We also found differences in event segmentation between the two viewing conditions. Having established a large and meaningful difference in comprehension of the clip, we then asked whether viewers in the Context and No-context conditions would show different eye movements, in terms of attentional synchrony and the probability of looking at the car (containing the bomb). To our surprise, that study has shown essentially the same pattern of results as the current study. Despite robust differences in comprehension using two different measures (inference and event segmentation), we found no difference in attentional synchrony, and only a small difference in eye movements to the car. Thus, we have found additional evidence for the two main claims of the current study, namely 1) attentional synchrony largely wipes out differences in eye movements caused by differences in comprehension (the *tyranny of film*), but 2) there can be small but subtle differences in eye movements due to comprehension if one carries out very fine-grained analyses.

### Challenges for current theories of scene perception

A considerable body of research has been carried out in the area of scene perception over the last 30 years (for reviews, see [69, 70]). However, a glaring hole in the body of scene perception research is the lack of research on the relationship between eye movements in scenes and higher-level narrative comprehension processes (but see [44]). Furthermore, given that the overarching goal of scene perception research is to explain perception of real-world scenes [69], an important new direction for such work is to account not only for the perception of static scenes, but also dynamic scenes in film and video. Thus, the current study represents an initial step in facing two challenges for scene perception research, namely accounting for the effects of higher-level narrative comprehension processes on lower-level processes such as eye movement control, and doing so within the context of film perception and comprehension.

### Challenges for current theories of film comprehension

We believe that theoretical accounts of film comprehension must explain when, why, and how eye movements and higher-level comprehension processes are coordinated [71]. The present results suggest that comprehension and eye movements can become relatively disassociated under the exogenous influence of intensified continuity film editing, but that the lack of such compositional constraints on attention in other visually presented narratives such as static images, plays, graphic novels and picture stories may allow greater expression of top-down endogenous factors.

A major question for any comprehensive theory of film perception and comprehension is how viewers process edits in narrative film [15, 43, 72]. One recent theory of film perception and comprehension in which eye movement control plays an integral part is AToCC. This theory explains how film editing manipulates overt attentional selection (saccades) in order to facilitate the process of maintaining cinematic continuity in the mind of the viewer across cuts a film. However, the current results pose a problem for AToCC as a theory of film comprehension. As currently formulated, AToCC prioritizes immediate spatiotemporal and object continuity over longer-term continuity dimensions involved in narrative film such as logical, causal, or emotional dimensions of a viewer’s event model [43, 47, 73, 74]. In this regard, it has been argued that “following the flow of visual continuity, a viewer is probably at the same time generating hypotheses about both the smaller and the larger stories within which the visual episodes fit” ([75], p. 38). Indeed, Smith [76] has argued that processes involved in *a posteriori* continuity, namely the psychological processes involved in restoring the perception of cinematic continuity following an unexpected transition produced by a cut, may also operate at these higher-levels, and may actually supersede the spatiotemporal level in importance for perceiving continuity (see also [77]). Importantly, an extension of AToCC to include narrative, causal and emotional dimensions would constitute a significant enlargement of the theory, which would require the foundation provided by existing theories of narrative comprehension (e.g., [47]).

## Conclusions

A key theoretical question in the study of visual attention is the degree to which eye movements are controlled exogenously by the stimulus versus endogenously by the viewer’s higher-level cognitive processes. The same question faces the fledgling theories of the perception and comprehension of film, namely what is the relationship between eye movements and higher-order comprehension processes, such as those involved in building mental models and using them to draw inferences? The current study has shown that such relationships can be easily obscured by the overwhelming attentional synchrony produced across viewers of Hollywood films. This is due to the exogenous attentional control exerted by the intensive continuity filmmaking style, which we refer to as the Tyranny of Film. Nevertheless, the current study has also shown that the endogenous higher-order comprehension processes involved in maintaining coherence throughout a cross-cutting shot sequence have subtle effects on viewers’ attentional synchrony and cognitive load, as evidenced by their eye movements. These findings point to the need for further work that bridges the gaps between theories of eye movement control in comprehension, scene perception, and film.
